# Pathways between Child Maltreatment, Psychological Symptoms, and Life Satisfaction: A Network Analysis in Adolescent Inpatients

**DOI:** 10.1007/s10802-024-01172-2

**Published:** 2024-01-30

**Authors:** David R. Kolar, Alessio Maria Monteleone, Giammarco Cascino, Sebastian Ertl, Adrian Meule, Silke Naab, Ulrich Voderholzer

**Affiliations:** 1 Department of Psychology, Clinical Child and Adolescent Psychology and Psychotherapy Institute of Psychology, University of Regensburg, Sedanstr. 1, 93055 Regensburg, Germany; 2 Department of Psychiatry, University of Campania L. Vanvitelli, Naples, Italy; 3 Department of Medicine, Surgery and Dentistry ‘Scuola Medica Salernitana’, University of Salerno, Salerno, Italy; 4 Department of Psychiatry and Psychotherapy, University Hospital, LMU Munich, Munich, Germany; 5 Schoen Clinic Roseneck, Prien am Chiemsee, Germany; 6 Department of Psychiatry and Psychotherapy, University Hospital of Freiburg, Freiburg, Germany; 7 Institute of Medical Psychology, Faculty of Medicine, LMU Munich, Munich, Germany

**Keywords:** Life satisfaction, Childhood trauma, Adolescents, Depression, Eating disorder

## Abstract

Child maltreatment is a risk factor for mental disorders and decreased life satisfaction during adolescence. We investigated whether child maltreatment would link to life satisfaction both directly and through psychological symptoms, whether these relations would change from admission to discharge after treatment, and which types of maltreatment, symptoms and facets of life satisfaction would be most influential in adolescent inpatients with internalizing mental disorders. *N* = 896 adolescent receiving inpatient psychotherapeutic treatment completed questionnaires on child maltreatment experiences, current psychopathology and subjective life satisfaction at admission and discharge (*n* = 765). Main diagnoses were affective (*n* = 322), eating (*n* = 447), obsessive–compulsive (*n* = 70) and anxiety disorders (*n* = 57). Network models of child maltreatment, psychopathology and life satisfaction nodes were estimated at admission and discharge and compared using network comparison tests. Potential causal shortest pathways were investigated using directed acyclic graphs.

Network models were stable with no significant differences between admission and discharge. Strongest nodes of each cluster were “emotional abuse” (child maltreatment), “worthlessness”, “thinking about dying” and “feeling lonely” (psychopathology) and “satisfied with life” (life satisfaction) at both admission and discharge. Emotional neglect showed direct connections to life satisfaction, indicating its relevance for therapeutic interventions. At both admission and discharge, “sexual abuse” indirectly predicted lower life satisfaction through psychological symptoms. In conclusion, child maltreatment is directly and indirectly connected to life satisfaction in adolescents with mental disorders. Emotional abuse and neglect were especially important in linking child maltreatment to life satisfaction and psychopathology.

**Supplementary Information:**

The online version contains supplementary material available at 10.1007/s10802-024-01172-2.

## Introduction

Maltreatment during childhood is a common experience for many children and adolescents across the world (Stoltenborgh et al., 2015). Child maltreatment is defined as “any act or series of acts of commission or omission by a parent or other caregiver that results in harm, potential for harm, or threat of harm to a child” (Arias et al., 2008, p. 19). According to Stoltenborgh et al. (2015), frequent types of maltreatment are physical, emotional and sexual abuse (prevalence proportions of 22.6%, 36.3% and 12.7% in self-report studies), and physical and emotional neglect (16.3% and 18.4%, respectively). Many individuals experience several types of child maltreatment. In a representative German sample, nearly 42% of the participants reporting *any* type of child maltreatment additionally reported *at least one other* form of maltreatment (Witt et al., 2017), which highlights the high co-occurrence of child maltreatment.

Child maltreatment has a tremendous negative impact on the lives of adolescents and adults: For example, it is an important risk factor for the onset and course of mental disorders (Chen et al., 2010; Lewis et al., 2019; McLaughlin et al., 2012). Furthermore, having experienced child maltreatment is also associated with lower health-related quality of life (HRQoL) during adolescence and adulthood (Cohrdes & Mauz, 2020; Davies et al., 2021; Weber et al., 2016; Witt et al., 2019). HRQoL is understood as a multidimensional construct that comprises perceived physical and psychological well-being as well as social functioning (Coghill et al., 2009; Herdman et al., 2002). Unsurprisingly, experiencing several types of child maltreatment was associated with lower HRQoL but only emotional abuse further decreased HRQoL when controlling for other types of child maltreatment (Cohrdes & Mauz, 2020; Witt et al., 2019).

Hence, specific interventions to reduce the impact of child maltreatment on HRQoL and psychological symptoms in children and adolescents are needed, especially for those with mental disorders. Although there is evidence for the effectiveness of cognitive behavior therapy (CBT) interventions on depression, anxiety and posttraumatic stress disorder symptoms in youth who experienced sexual abuse (see e.g. Macdonald et al., 2016; Wethington et al., 2008), evidence is weaker for the effectiveness of CBT on other forms of child maltreatment including physical abuse and neglect (Lorenc et al., 2020; Macdonald et al., 2016). Remarkably, most treatment studies for child maltreatment did not consider HRQoL as a potential outcome beyond symptom improvement. In general, investigating improvements in HRQoL as a desirable outcome of psychological and psychiatric treatments has received increased research interest within the last years (Thapa Bajgain et al., 2023), as it accounts for the psychosocial impairments associated with mental disorders (Dey et al., 2012; Sharpe et al., 2016). Especially measuring perceived life satisfaction, a HRQoL dimension of how people assess their general well-being, might be promising. However, only few studies investigated HRQoL in youth who experienced child maltreatment (Weber et al., 2016). It is therefore unclear whether improving mental health in adolescents, who experienced child maltreatment, would immediately translate into higher HRQoL or if these adverse experiences continue to impact after treatment for mental disorders.

Most of the studies that investigated the impact of child maltreatment on mental health, however, were conducted using composite scores of symptoms or investigated differences between mental disorders. This assumes that mental disorders are natural discrete diagnostic entities which are sustained by a group of symptoms (Maj, 2018), the so-called latent-disease model (i.e., a latent depression factor results in observable depressive symptoms). However, this assumption is challenged when considering important transdiagnostic psychopathological processes (e.g. rumination; Cludius et al., 2020), diagnostic overlap in symptoms across different disorders (Borsboom et al., 2011) and consequently high comorbidity between mental disorders (e.g. shared symptoms of restlessness and concentration problems might explain comorbidity of generalized anxiety and major depressive disorders; Kaiser et al., 2021; Zbozinek et al., 2012). In addition, different types of child maltreatment experiences are most likely having distinct impact on HRQoL and might be connected to specific psychological symptoms, which themselves most arguably have unique relations to HRQoL. Therefore, advanced methodological approaches are needed to simultaneously investigate complex systems of child maltreatment experiences, psychological symptoms and life satisfaction.

### Network Theory of Mental Disorders

A modern view on mental disorders accounting for the above-mentioned problems in the latent-disease model is the network theory of mental disorders (Borsboom, 2017). Network theory understands psychopathology as a complex system of interrelated symptoms activated by external events (i.e., experiences of abuse or neglect), which themselves activate other symptoms and, individually and in sum, impacting HRQoL of an individual (Borsboom, 2017). Thus, in a network model, symptoms themselves are considered to constitute a disorder rather than being caused by common latent factors (Borsboom, 2017; Cramer et al., 2010; Kendler et al., 2011), and mental disorders merely reflect stable states of symptom networks (Cramer et al., 2016). In addition, treatment effects can then be seen and modelled as “shocks” to specific interactions between network elements (e.g., breaking the link between sleep disturbances and negative mood might switch a network from a depressed into a non-depressed state; see Cramer et al., 2016). This view allows for better understanding individual differences, comorbidity and transdiagnostic processes in psychopathology.

### Network Analysis – a Modern Approach for Investigating Psychopathology

Simultaneously, a statistical method called network analysis has been widely introduced to investigate mental disorders as complex systems (for an overview see McNally, 2016). In cross-sectional data, network analysis allows for estimating reciprocal (i.e., undirected) relationships between “nodes” (i.e., variables such as symptoms, external events) simultaneously, which are called edges and are conditioned on all other edges. Researchers can then inspect centrality indices that inform about how important (i.e., how well-connected to other nodes) and influential (i.e., how strong are connections to other nodes) a given node is. It is also possible to compare network models, for example pre- to post-treatment, and inspect changes in the overall structure (changes in connectivity after intervention) and specific edges (e.g., is the relationship between sleep disturbances and negative mood maintained after treatment). Using Bayesian networks, it is also possible to compute a directed acyclic graph (DAG) that can unravel directed and potentially causal relationships from one node to another (for an introduction see Briganti et al., 2022).

Network analyses have already revealed pathways of associations between child maltreatment and psychopathology that may reflect underlying causal processes. For example, Fritz et al. (2018) found a dysfunctional resilience system in adolescents who were exposed to adverse circumstances in childhood. Results suggest that some resilience factors attenuate others and are generally more directly related to current stress. Another study highlighted especially emotional maltreatment to be most influential in the network and connected other types of child maltreatment to well-being in adulthood (Volgenau et al., 2022). Su et al. (2023) found evidence for specific HRQoL connectivity patterns associated with experienced child maltreatment in adults. In addition, they identified emotional neglect and emotional abuse as types of child maltreatment with the greatest impact on HRQoL. Consistent with these findings, emotional abuse appears to play an important role in activating depressive and suicidal symptoms in adults with major depressive disorder (Zhou et al., 2022).

Therefore, network analysis might also help identifying potential mechanistic pathways from child maltreatment through direct and symptom-mediated pathways to HRQoL in adolescents with mental disorders. In addition, comparing network structures of child maltreatment, psychological symptoms and life satisfaction before and after intensive treatment might identify potential novel treatment targets. For example, identifying specific symptoms connecting child maltreatment to HRQoL in networks before and after treatment might be promising treatment candidates. It is of great importance to further improve treatment for this patient group considering that adolescent inpatients who experienced child maltreatment have a worse treatment outcome compared to those who did not (Voderholzer et al., in prep).

### The Current Study

Previous studies highlighted potential associations between child maltreatment and HRQoL, yet clear pathways have not been established. Specifically, it is still unclear whether these pathways include psychological symptoms. Furthermore, most studies that examined child maltreatment using network analysis included adults. In this exploratory study, we investigated whether child maltreatment would connect to the HRQoL associated dimension of life satisfaction both directly and through psychological symptoms, whether these relations would change from admission to discharge after inpatient psychotherapeutic treatment, and which types of maltreatment, symptoms and facets of life satisfaction would be the most important links^1^. For this study, we rely on self-reported child maltreatment as other sources are often unavailable in treatment settings. While some studies note an underreporting of child maltreatment in self-reports (with almost zero false reports of child maltreatment; see Fergusson et al., 2000), self-reports are generally considered to be sufficiently reliable over time and stages of illness (Goltermann et al., 2023; Pinto et al., 2014). In addition, self-reports may also account for the subjective component of severity in a dimensional manner. We used network analysis to achieve the following three aims:

#### Aim 1

To identify “key symptoms” in associations between five types of child maltreatment, psychological symptoms and life satisfaction at admission and discharge.

#### Aim 2

To explore potential causal relationships between child maltreatment, life satisfaction and psychological symptoms.

#### Aim 3

To identify changes in associations between child maltreatment, psychological symptoms and life satisfaction over the course of inpatient treatment.

## Methods

### Participants and Procedure

Data from *N* = 1409 adolescents aged 12 to 19 years were recorded who were admitted to inpatient treatment between August 2015 and January 2021 at the Schoen Clinic Roseneck, a large inpatient treatment center in Bavaria, Germany. Patients were treated in a psychosomatic-psychotherapeutic hospital, which encompasses a multimodal treatment in a multi-professional team. Indication for psychosomatic-psychotherapeutic treatment covers most internalizing disorders (e.g., affective, anxiety, or eating disorders). In contrast to inpatient psychiatric treatment, acute suicidality and psychotic disorders are contraindications (for a more detailed description see Zipfel et al., 2016). Indications for inpatient instead of outpatient treatment were severity of symptoms, serious comorbidity or lack of response to outpatient treatment. Due to the treatment focus of the Schoen Clinic Roseneck, patients with eating disorders were also admitted in case of rapid weight loss or clinically significant underweight. For primary diagnosis included in the sample and their descriptive statistics see Table S1 in the electronic supplement. Patients with a primary diagnosis of post-traumatic stress disorder were excluded from our study. Patients were diagnosed by experienced clinicians with training in child and adolescent psychiatry or psychotherapy at admission according to the International Statistical Classification of Diseases, 10th revision (World Health Organization, 2004). All patients received intensive CBT-oriented multimodal inpatient treatment, including individual psychotherapy and group therapy sessions twice per week. Additional disorder-specific treatments were offered (e.g., exercise therapy and body image exposure for eating disorders or exposure with response prevention for obsessive–compulsive disorder [OCD]). Adolescents were asked to fill in questionnaires electronically or paper-based at admission and discharge as part of the routine assessment procedure. Adolescents were excluded if they received a different main diagnosis than any affective disorder, anxiety disorder, anorexia nervosa, bulimia nervosa or OCD, as these were the main diagnostic groups during the assessment period, resulting in *N* = 1290 eligible participants. Adolescents were further excluded if they had missing values in the Childhood Trauma Questionnaire (CTQ) subscales, selected Brief Symptom Inventory (BSI) or Satisfaction With Life Scale (SWLS) items due to the necessity of complete case data for estimating network models. The final datasets consisted of *n* = 896 adolescents for the admission network model, *n* = 765 adolescents for the discharge model and *n* = 635 adolescents for comparing networks. Exclusion mainly occurred due to either entirely missing BSI and SWLS (*N* = 165; 12.8%) *or* the last 14 items of the CTQ due to a technical error (*N* = 131; 10.16%). When comparing excluded and included patients, excluded patients were marginally younger (included: *M* = 16.03 years, *SD* = 1.25; excluded: *M* = 15.86 years, *SD* = 1.26; *t*[744.59] = 2.26, *p* = .0241), but they did not differ regarding gender (*χ²*[1] = 1.29, *p* = .26) or primary diagnosis (*χ²*[4] = 7.48, *p* = .11). Comparing data from patients that responded to BSI items as well as SWLS items at admission and discharge to those that did only at admission revealed a statistically significant but clinically negligible difference in the item mean score of the 30 BSI items that were selected for network analyses, but no difference for SWLS items (see Table S2). CTQ sum scores also differed between patients with complete data at admission and discharge compared to those that missed at least one BSI or SWLS item at discharge with a small effect size. The study was approved by the institutional review board of the ethics committee of the medical faculty at the Ludwig-Maximilians-University Munich, Germany (No.21-0606). In accordance with the guidelines of the ethics committee, retrospective analyses of completely anonymized data do not require informed consent.

### Measurements

#### Childhood Trauma Questionnaire (CTQ)

Abuse and neglect during childhood was assessed retrospectively at admission using the 28-item CTQ short-form (Bernstein et al., 2003; German version: Klinitzke et al., 2012). It contains five subscales consisting of five items each that assess different types of abuse and neglect: emotional abuse, emotional neglect, physical abuse, physical neglect and sexual abuse. An additional three-item scale assesses minimization/denial of abuse or neglect and was not further assessed in this study. The CTQ is answered on a five-point scale ranging from 1 – *not at all* to 5 – *very often*. Scale scores are sum scores with values between 4 and 25 (up to one item can be missing for a scale to be computed). Categorical interpretations of child maltreatment severity were computed for descriptive purposes only (four categories: *none to minimal*, *slight to moderate*, *moderate to severe*, *severe to extreme*), with child maltreatment presumed if sum scores fall into the “slight or moderate” category or higher (see Häuser et al., 2011, for details). Validity was corroborated with positive correlations with anxiety and depressiveness as well as negative correlation with satisfaction with life in a representative German sample (Klinitzke et al., 2012). In our study, internal consistency was good with 0.80 ≤ McDonald’s *ω* ≤ 0.91, except for the physical neglect subscale (*ω* = 0.48).

#### Satisfaction with Life Scale (SWLS)

We used the SWLS (Diener et al., 1985; German version: Glaesmer et al., 2011) to assess subjective life satisfaction at admission and discharge. The SWLS contains five items assessed on a seven-point scale from 1 – *strongly disagree* to 7 – *strongly agree*. Composite scores are sum scores ranging from 5 to 35. Higher sum scores indicating higher life satisfaction. Positive indicators for convergent validity such as a positive correlation with social support as well as a negative correlation with depressiveness were previously found in a large German sample (Glaesmer et al., 2011). Internal consistency was good with McDonald’s *ω* = 0.83 at admission and ω = 0.88 at discharge. In this study, we relied on item-level scoring of the SWLS.

#### Brief Symptom Inventory (BSI)

The BSI measures psychopathology (Derogatis & Melisaratos, 1983; Franke, 2000) by assessing 53 frequent psychological symptoms. Adolescents indicated how much they were bothered by these symptoms within the last week on a five-point scale from 0 – *not at all* to 4 – *very much*. A subset of 30 items was selected for the network analysis, based on criteria described in detail below. To compare changes in psychopathology from admission to discharge, the global severity index—an item mean score based on all 53 items—was computed, which correlated substantially with the longer Symptom Check List global score in the original studies (Derogatis & Melisaratos, 1983; Franke, 2000). Internal consistency of the total scale was excellent with McDonald’s *ω* = 0.96 at admission and *ω* = 0.97 at discharge.

### Statistical Analyses

#### Item Selection for Admission and Discharge Networks

CTQ subscale sum scores were included in the network analysis. CTQ scores were only assessed at admission as they referred to experiences during childhood. All SWLS items were selected. Item selection for psychological symptoms was based on (a) theoretical importance in adolescence, (b) non-collinearity of items at admission and (c) inspecting histograms of the items (details available in Table S3 and Figure S1). Thus, we excluded items that were not frequently endorsed by adolescents or of less importance (e.g., item 41 “urge to break things”). Items 2 (“fainting”), 7 (“chestpain”) and 33 (“feeling numb”) were identified as colinear with item 30 (“hot and cold spells”) with less than 25% significantly different correlations to all other variables by the *goldbricker* function of the *networktools* package, v1.3.0 (Jones, 2021), and only item 30 was retained. Finally, 30 items were included in network models (see Table 1 for item list).

**Table 1:** Overview of network nodes including corresponding scale, item description and mean (SD) at admission and discharge

Nodes	Scale	Item description	Admission		Discharge	
			M	(SD)	M	(SD)
emoabu	CTQ	Emotional abuse	9.43	(4.64)	9.14	(4.44)
emoneg	CTQ	Emotional neglect	10.01	(4.28)	9.69	(4.13)
physabu	CTQ	Physical abuse	5.60	(1.72)	5.55	(1.75)
physneg	CTQ	Physical neglect	6.57	(2.17)	6.49	(2.10)
sexabu	CTQ	Sexual abuse	5.54	(2.05)	5.46	(1.73)
bsi01	BSI	Nervousness	1.98	(1.22)	1.47	(1.15)
bsi06	BSI	Feeling easily irritated	2.02	(1.27)	1.45	(1.21)
bsi09	BSI	Suicidal thoughts	0.85	(1.09)	0.63	(0.95)
bsi10	BSI	Distrust in people	1.53	(1.30)	1.22	(1.20)
bsi11	BSI	Poor appetite	1.68	(1.39)	1.06	(1.18)
bsi13	BSI	Uncontrollable temper outbursts	1.94	(1.34)	1.22	(1.24)
bsi14	BSI	Feeling lonely in company of other people	2.23	(1.40)	1.67	(1.33)
bsi15	BSI	Unable to complete tasks	2.01	(1.38)	1.48	(1.27)
bsi16	BSI	Feeling lonely	2.38	(1.31)	1.75	(1.33)
bsi17	BSI	Feeling blue	1.75	(1.29)	1.19	(1.19)
bsi18	BSI	Loss of interest	1.82	(1.35)	1.05	(1.14)
bsi19	BSI	Feeling fearful	1.42	(1.26)	0.97	(1.16)
bsi21	BSI	Feeling disliked by others	1.95	(1.43)	1.58	(1.35)
bsi22	BSI	Feelings of inferiority	2.22	(1.41)	1.76	(1.39)
bsi23	BSI	Nausea	1.44	(1.32)	1.02	(1.13)
bsi24	BSI	Feeling watched or talked about by others	1.82	(1.37)	1.22	(1.20)
bsi25	BSI	Insomnia	1.77	(1.38)	1.12	(1.22)
bsi27	BSI	Decision-making difficulties	2.18	(1.31)	1.59	(1.26)
bsi30	BSI	Hot or cold spells	1.33	(1.27)	0.94	(1.09)
bsi35	BSI	Hopelessness	2.13	(1.36)	1.46	(1.31)
bsi36	BSI	Concentration problems	1.94	(1.38)	1.36	(1.24)
bsi37	BSI	Weekness in body parts	1.18	(1.25)	0.72	(0.99)
bsi38	BSI	Feeling tense	2.04	(1.28)	1.76	(1.19)
bsi39	BSI	Thinking about dying	1.23	(1.31)	0.92	(1.17)
bsi43	BSI	Feeling uneasy in crowds	1.49	(1.46)	0.90	(1.21)
bsi44	BSI	Never feeling close to others	1.55	(1.40)	1.23	(1.32)
bsi49	BSI	Restlessness	1.06	(1.21)	0.84	(1.08)
bsi50	BSI	Worthlessness	2.18	(1.46)	1.59	(1.40)
bsi52	BSI	Guilt	2.15	(1.42)	1.64	(1.32)
bsi53	BSI	Idea that something is wrong with one’s mind	1.80	(1.42)	1.27	(1.36)
swls01	SWLS	My life is close to ideal	2.84	(1.59)	3.55	(1.60)
swls02	SWLS	Excellent conditions of my life	4.00	(1.80)	4.64	(1.52)
swls03	SWLS	Satisfied with my life	2.74	(1.58)	3.79	(1.68)
swls04	SWLS	Achieved important things in life	3.02	(1.60)	3.51	(1.62)
swls05	SWLS	Would change nothing if could live my life again	2.62	(1.65)	3.01	(1.73)

Note: BSI: Brief Symptom Inventory; CTQ: Childhood Trauma Questionnaire; SWLS: Satisfaction With Life Scale. CTQ was only administered at admission. Thus, CTQ scores reflect mean and SD of the subsamples with BSI and SWLS data at admission or discharge

#### Network Estimation

We constructed separate networks at admission and discharge through *R*, v4.0.3 (R Core Team, 2022) and *RStudio*, v1.3.1093 (RStudio Team, 2022), using the *qgraph* package, v1.6.9 (Epskamp et al., 2012). To account for non-normality, we applied a nonparanormal transformation using the *huge* package, v1.3.5 (Zhao et al., 2011).

We fitted Gaussian Graphical Models (GGM) to our data. Within these models, CTQ subscales, SWLS items and BSI symptoms are represented as nodes, whereas edges between two nodes are partial correlations conditioned on all other nodes. Associations are undirected, but CTQ nodes temporally precede all other nodes. Missing edges indicate independence of two nodes after conditioning on all other nodes. We applied a Least Absolute Shrinkage and Selection Operator (LASSO) regularization to shrink small partial correlations and set them to zero (Friedman et al., 2008). The Extended Bayesian Information Criterion, a parameter to set the degree of regularization applied to sparse correlations, was set to γ = 0.5 (Chen & Chen, 2008).

#### Network Inference and Stability

We computed centrality indices for both networks to investigate the network structure, including node strength (sum of all edges of a given node to all other nodes) and expected influence (summed weight of a node’s edges shared with remaining nodes). We did not compute betweenness and closeness as recent research indicates that these centrality measures are unsuitable for psychopathological networks (Bringmann et al., 2018). We estimated network stability of centrality measures and the accuracy of edge-weights by drawing bootstrapped confidence intervals using the nonparametric bootstrapping function (nboots = 2500) of the *bootnet* package, v1.4.3 (Epskamp et al., 2018), and report correlation stability coefficients (CS) for centrality measures. We followed recommendations and only report centrality measures above the threshold of 0.5 (Epskamp et al., 2018).

#### Bayesian Network-based DAGs

To investigate potentially causal pathways from childhood maltreatment to life satisfaction, Bayesian networks to model DAGs were estimated. We followed the procedure described by McNally et al. (2017), using a machine-learning algorithm (score-based hill-climbing algorithm) implemented in the R package *bnlearn* (Scutari, 2010). Network structure has been learned by the algorithm through iteratively adding, removing, and reversing edges achieving an optimal goodness-of-fit target score (BIC, *Bayesian information criterion)*. We bootstrapped *N* = 10,000 network-samples for networks at admission and discharge to determine stability of parameters. The final admission and discharge networks were calculated by averaging bootstrapped networks using an empirical approach suggested by Scutari and Nagarajan (2013). Edges between variables which exceeded the empirically determined threshold were retained in the DAG. Additionally, direction of edges was included if it appeared in at least 51% of the bootstrapped samples. Edge strength was defined as the rate of appearing edges in the bootstrapped samples (Csardi & Nepusz, 2006), depicted via thickness of edges in the graph (e.g., relatively thick edges indicate higher level of replications). Directed edges from life satisfaction (SWLS) and psychological symptoms (BSI) to CTQ-subscales were excluded (blacklisted) from analyses considering the temporal precedence of child maltreatment. Shortest pathways between CTQ and SWLS nodes, indicating direct and indirect pathways from childhood traumatic experiences to life satisfaction, were computed using Dijkstra’s algorithm (Dijkstra, 1959) implemented in the *igraph* package, v1.4.1 (Csardi & Nepusz, 2006).

#### Network Comparisons between Admission and Discharge Networks

Finally, we compared undirected GGM-networks for admission and discharge with the network comparison test (NCT) using the *NetworkComparisonTest* package, v2.2.1 (van Borkulo et al., 2021). The NCT is a permutation-based hypothesis test for comparing GGMs of dependent samples and assesses the difference between two networks on invariance measures (network structure, global strength, and edge invariance). Differences in edges were inspected using post-hoc comparisons. Changes in the estimated BNs were investigated descriptively, comparing shortest pathways in DAGs at admission and discharge.

#### Availability of Data and Code

Analyses were not preregistered. Anonymized data and code to reproduce the network analyses are available at https://osf.io/pyke9/.

## Results

896 (816 female, 80 male) adolescent inpatients aged 12 to 19 years (*M* = 16.03 years, *SD* = 1.25; 78% aged 15 to 17 years) provided data at admission, and 765 (703 female, 62 male) adolescents again at discharge. Of those, 322 (35.93%) were diagnosed with affective disorders, 447 (49.89%) with eating disorders, 70 (7.81%) with OCD and 57 (6.36%) with anxiety disorders as primary diagnosis. On average, adolescents improved from admission to discharge on psychological symptoms and life satisfaction (both *p* ≤ .001; see Table S4) and were treated for an average of 92 days (*SD* = 50.26; IQR: 56–121 days). Applying a cut-off score, 22.65% of the adolescents reported having experienced emotional abuse, 15.63% being emotionally neglected, 4.13% being physically abused, 8.37% having experienced physical neglect and 7.25% being sexually abused during their childhood. Significant differences between diagnostic groups regarding age, length of inpatient treatment, childhood trauma, symptom severity and life satisfaction emerged (see Table S1, also for additional demographic data).

### Aim 1: Network Estimation at Admission and Discharge

**Fig. 1:**
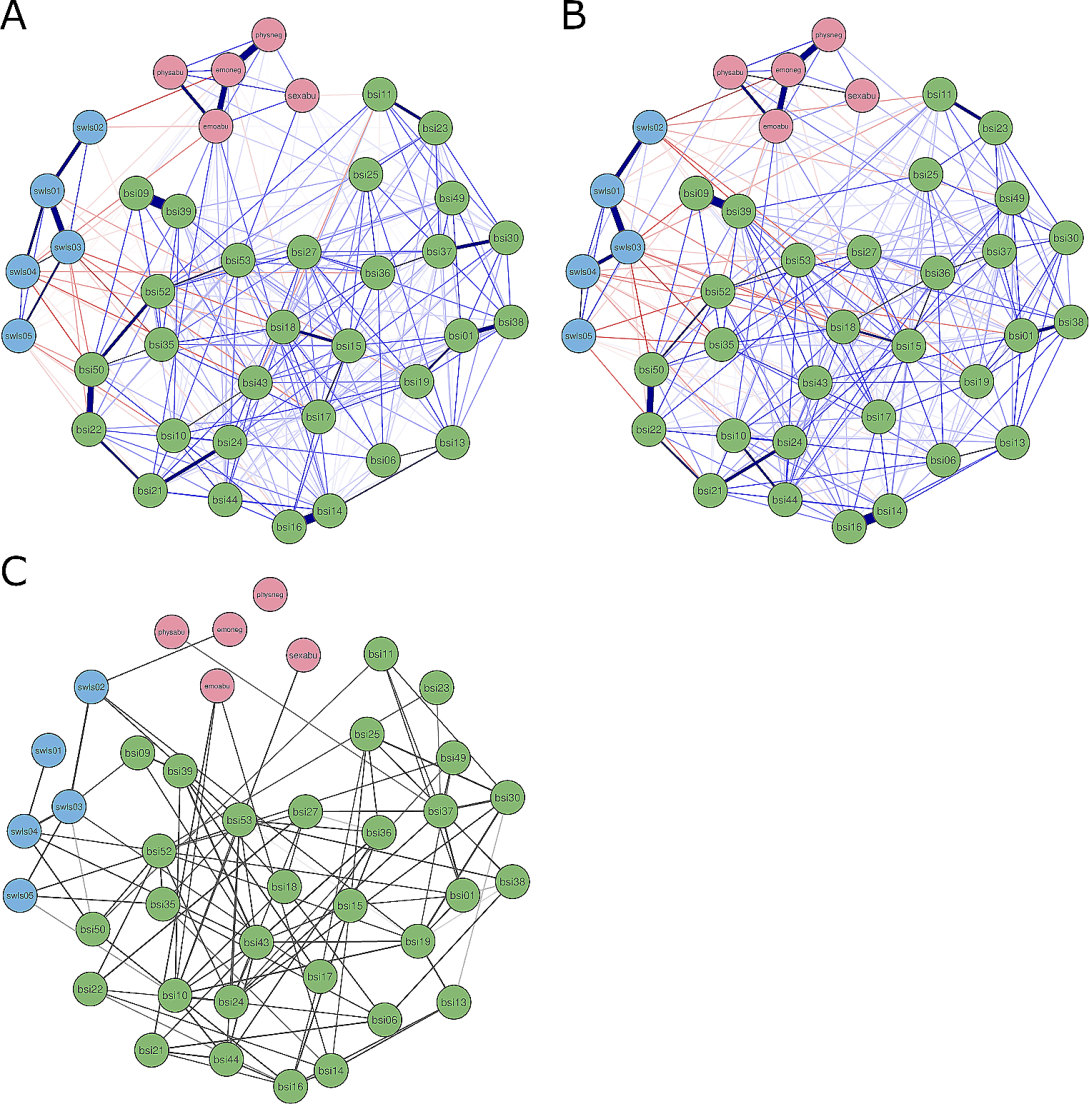
Estimated networks. (A) admission (*n* = 896) and (B) discharge (*n* = 765) networks. Nodes (circles) represent symptoms, maltreatment types and facets of life satisfaction. Width and saturation of an edge (lines connecting nodes) represent the strength of the correlation, whereas color indicates positive (blue) or negative (red) correlations. Node color represents belonging to either symptom, child maltreatment or life satisfaction communities. In the variability network (C), edges represent the standard deviation of edges between two specific nodes across admission and discharge networks. See Table 1 for full node descriptions and communities (i.e., scales). A high-resolution version of the figure can be found here: https://osf.io/pyke9/

We constructed two networks to highlight associations between childhood abuse and neglect (CTQ subscales, red nodes), psychological symptoms (30 items selected from BSI, green nodes) and life satisfaction (SWLS items, blue nodes) separately at admission (Fig. 1A) and discharge (Fig. 1B). When inspecting the networks, the discharge network appears denser (i.e., more strongly connected). Strong connections (i.e., greater partial correlation coefficients) between *physneg* and *emoabu* with *emoneg* emerged, highlighting their frequent co-occurrence during childhood. Strong connections between *swls02* and *swls03* with *swls01* emerged in both networks, whereas other edges between life satisfaction nodes differed in strength between admission and discharge. Strongest edges of symptoms unsurprisingly emerged between *bsi09* and *bsi39* (suicidal thoughts and thoughts about dying), *bsi14* and *bsi16* (both facets of feeling lonely), as well as *bsi22* and *bsi50* (feeling inferior and worthlessness).

#### Network Stability and Inference

**Fig. 2:**
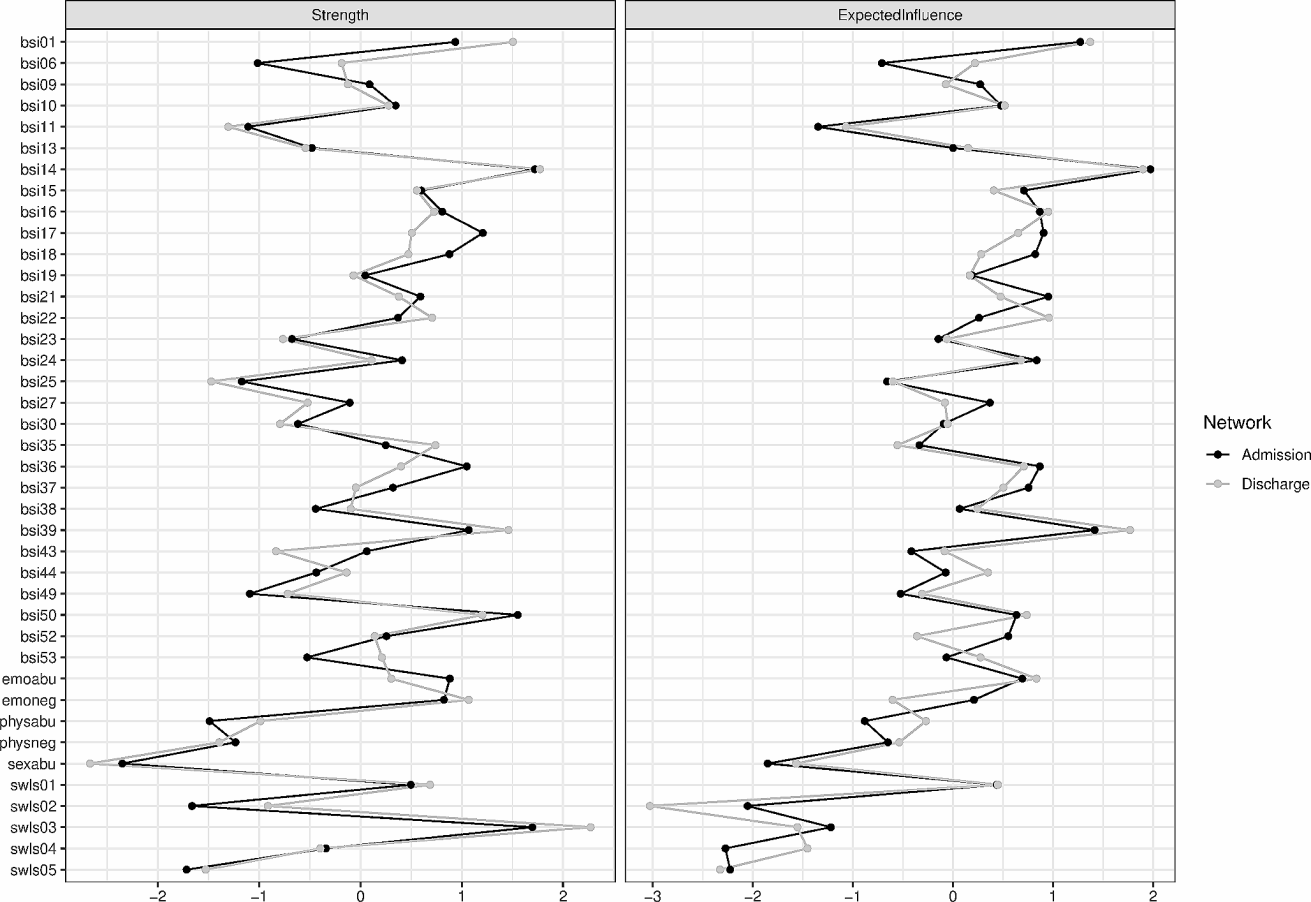
Standardized node strength (sum of all edges of a given node to all other nodes) and expected influence centrality (summed weight of a node’s edges shared with remaining nodes) for admission and discharge networks. A high-resolution version of the figure can be found here: https://osf.io/pyke9/

Networks were highly stable at admission and discharge regarding node strength and expected influence (all CS > 0.75). Figure 2 shows centrality measures for both networks. Node strength was highest for *emoabu* from child maltreatment (*emoneg* at discharge), *bsi50* (worthlessness), *bsi39* (thinking about dying) and *bsi14* (“feeling lonely”) from psychological symptoms, and *swls03* (“satisfied with life”) from life satisfaction items at admission and discharge. Consistently low node strength was observed for *sexabu*, *swls05* (“live same life again”), *bsi11* (“poor appetite”) and *bsi25* (“insomnia”). Finally, the overall expected influence of nodes did not vary much from admission to discharge, with *swls01*, *emoabu*, *bsi39* (“thinking about dying”) and *bsi14* (“feeling lonely”) showing largest expected influence.

### Aim 2: Prediction of Life Satisfaction through Child Maltreatment Experiences (DAG Analysis)

**Fig. 3:**
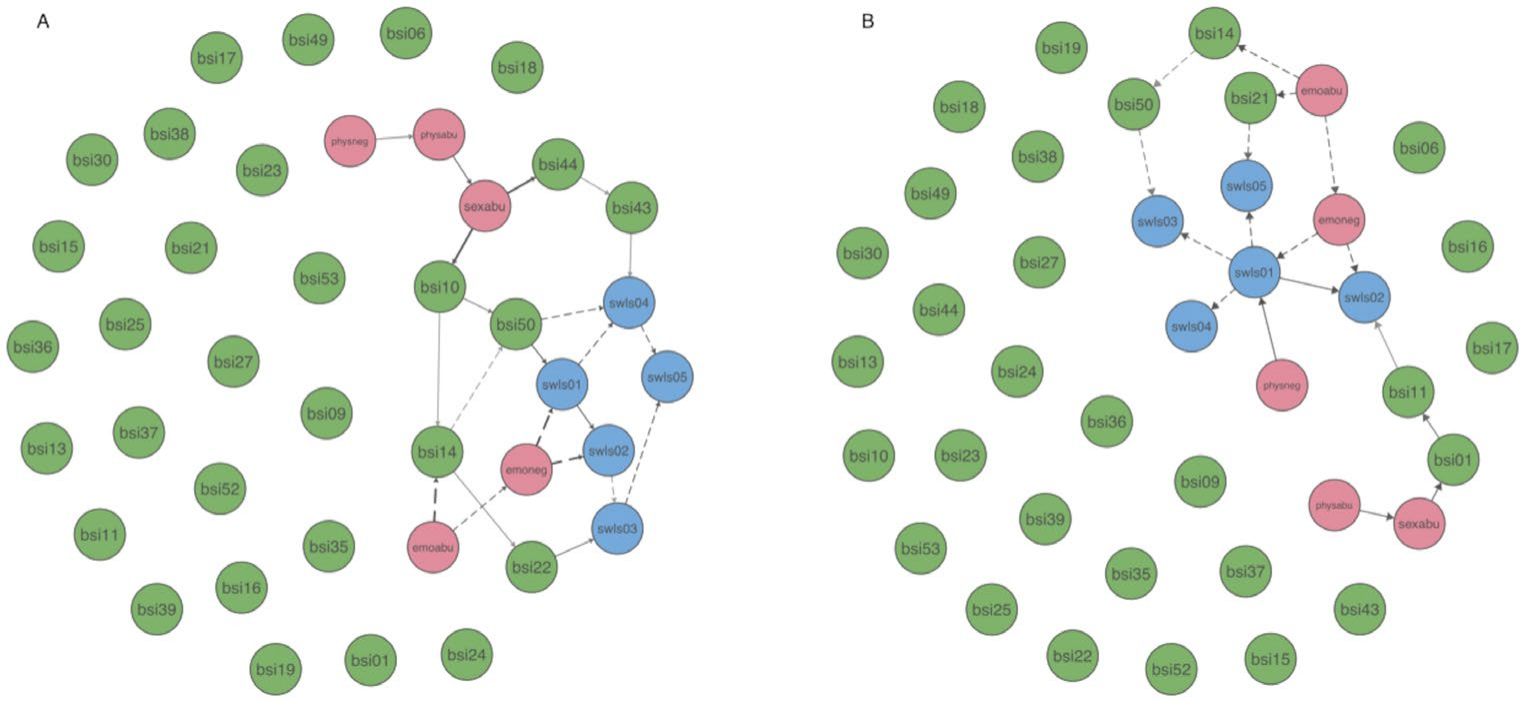
Shortest directed pathways from child maltreatment to life satisfaction nodes (circles) at (A) admission (*n* = 896 patients) and (B) discharge (*n* = 765 patients) based on directed acyclic graphs (DAGs) of *N* = 10,000 bootstrapped samples. Node color represents belonging to either symptom, child maltreatment or life satisfaction communities. Edges (arrows connecting nodes) represent potentially causal pathways between nodes exceeding the empirically determined threshold were retained; direction of edges was included if present in at least 51% of the bootstrapped samples. Edge thickness represents confidence in prediction. DAGs are combined for all forms of child maltreatment (pathways from emoabu–emoneg to SWLS are represented by dashed lines, paths associated with sexual abuse are shown with solid lines). A high-resolution version of the figure can be found here: https://osf.io/pyke9/

Figure 3 represents the shortest directed pathways from child maltreatment to life satisfaction nodes as they emerged in the DAGs for admission and discharge (full DAGs are available in supplementary materials Figure S2). Edges are displayed when reaching the threshold of being directed in the same direction in at least 49.97% in admission and 50.31% in discharge bootstrapped samples. Thickness of edges represents confidence in prediction of the direction emerged from bootstrapped samples. First, direct pathways to life satisfaction nodes emerged only for *emoneg*, indicating that higher values in *emoneg* predicted lower values in both *swls01* (“my life is close to ideal”) and *swls02* (“excellent condition of my life”) in the admission network (Fig. 3A). The path from *emoneg* through *swls01* then further proliferates into *swls03* (“satisfied with my life”) and *swls04* (“achieved important things in my life”). *Emoneg* itself was predicted by *emoabu*, suggesting that some effects of experienced emotional abuse on life satisfaction are mediated by emotional neglect (shortest paths to *swls01*, *swls02*, *swls03* and *swls05*). In addition, an indirect prediction of *swls04* emerged through connections from *emoabu* to *bsi14* (“feeling lonely in company of other people”) and *bsi50* (“Worthlessness”). A second main branch showed three indirect shortest pathways to life satisfaction via *sexabu*, First, sexual abuse predicted *bsi10* (“distrust in people”) which was connected to *bsi50* and then to *swls01*, followed by *slws02.* A second path emerged that connected *sexabu* again through *bsi10* but also *bsi14* and *bsi22* (“feeling inferior to others”) to *swls03*. This suggests that ”distrust in people” could be a key symptom between child maltreatment experiences and life satisfaction. Finally, *sexabu* predicted *swls04* followed by *slws05* through *bsi44* (“Never feeling close to others”) and *bsi 43* (“feeling uneasy in crowds”). As *sexabu* was predicted by *physabu* which was predicted *physneg*, the pathways from *sexabu* also proliferate effects of physical child maltreatment to life satisfaction.

In the discharge DAG, *swls01* and *swls02* were also predicted by *emoneg* (Fig. 3B). In addition, *swls01* was directly predicted by *physneg*. *Swls01* then proliferates effects further to other life satisfaction nodes. *Emoabu* predicted *swls05* (“Would change nothing if I could live my life again”) through *bsi21* (“Feeling disliked by others”) and *swls03* (“satisfied with my life”) through *bsi14* and *bsi50*. Physical abuse predicted sexual abuse, which showed indirect paths to only *swls02* through *bsi01* (“nervousness”) and *bsi11* (“poor appetite”). *Physneg* predicted *swls01*, which further proliferated effects into other life satisfaction domains.

### Aim 3: Comparisons of Undirected Overall Network Structures and Directed Shortest Pathways from Child Maltreatment to Life Satisfaction between Admission and Discharge

Centrality measures at admission and discharge were highly correlated (strength: *r* = .90; expected influence: *r* = .84). We used the NCT to compare joint network structures of child maltreatment, psychological symptoms and life satisfaction at admission and discharge based on *n* = 635 adolescents with data for both assessments. The structure of the undirected partial correlation networks appeared identical (*M* = 0.15, *p* = .126) and with similar overall connectivity (*S* = 0.03, *p* = .901) at admission and discharge. We also tested for differences of edges between child maltreatment and life satisfaction nodes, as network invariance might be overestimated as CTQ nodes were only measured once. When applying Bonferroni-Holm correction for edge invariance tests, only the edge between *physabu* and *swls01* (*p* = .002) was significantly different between admission and discharge, which is further highlighted by emerging as a directed edge only in the discharge DAG. Figure 1C depicts the admission-to-discharge variability network. Standard deviations were small to negligible. The most variable edges were *bsi30* – *bsi37* (*SD* = 0.10) and *swls03* – *swls04* (*SD* = 0.09).

Regarding the directed shortest pathways from child maltreatment to life satisfaction, descriptive comparisons of DAGs suggest that direct pathways were mostly stable, underlining the central role of *emoneg*. The indirect link between *sexabu* and life satisfaction through *bsi10* was unstable and changed completely from admission to discharge, such that at the end of treatment now only *swls02* was affected by *sexabu*, mediated trough *bsi01* and *bsi11*.

## Discussion

We used network analyses to investigate the interrelations of experiences of child maltreatment, psychological symptoms, and life satisfaction in adolescents with mental disorders currently receiving inpatient treatment. Partial correlation networks were highly stable and reproducible at admission and discharge, with emotional neglect, “worthlessness”, “thinking about dying”, “feeling lonely” and “satisfied with life” being the most central nodes of either childhood maltreatment, psychological symptoms or life satisfaction nodes at both admission and discharge. We estimated DAGs using Bayesian networks at admission and discharge to further investigate potential causal connections between child maltreatment and life satisfaction. The DAGs support our previous findings, identifying emotional neglect as directly connected to several life satisfaction nodes and additionally connecting emotional abuse to life satisfaction. Except for indirect paths from sexual abuse and emotional abuse to life satisfaction, no potentially causal shortest pathways from child maltreatment through psychological symptoms to life satisfaction were found at admission.

Looking at changes from admission to discharge, partial correlation networks were not statistically significantly different. Despite DAGs showing some differences between admission and discharge, main links between child maltreatment and life satisfaction were similar, especially regarding the importance of emotional neglect. However, new pathways to life satisfaction emerged for physical neglect (now directly connected). Indirect shortest pathways to life satisfaction through psychological symptoms emerged only for emotional and sexual abuse. However, they were not stable across admission and discharge.

Our findings support and extend previous findings using network analyses in the field of child maltreatment. For example, Breuer et al. (2020) pointed out that sexual abuse is more closely connected to DSM–5 diagnoses of mental disorders compared to other forms of child maltreatment. This is reflected in our finding showing that sexual abuse was only exclusively connected to life satisfaction through psychological symptoms. Our findings are also in line with recent literature highlighting child maltreatment and adverse childhood experiences as risk factors for a multitude of life problems in adolescence and adulthood extending beyond mental disorders (Widom, 2014). The finding that these experiences are directly linked to the HQoL associated dimension of life satisfaction further underlines the detrimental effects of child maltreatment, even when controlling for psychological symptoms in a sample of adolescents currently undergoing inpatient treatment for these symptoms. Emotional abuse and neglect were the most important types of child maltreatment in our sample, as they predicted lower life satisfaction both through direct and indirect connections. These types of maltreatment were also most common in our sample, which might partially explain less robust findings for other types of maltreatment. In line with similar findings from other studies (Monteleone et al., 2019, 2021), this further supports that (a) physical maltreatment and sexual abuse are often accompanied by emotional maltreatment and (b) especially emotional maltreatment is connected to later low HRQoL and psychological symptoms (Hagborg et al., 2017; Taillieu et al., 2016). Understanding mental disorders as complex systems (Borsboom, 2017), child maltreatment may work as an exogenous trigger that promotes long-lasting changes in neurocognitive and emotion regulation processes (Cowell et al., 2015; Gruhn & Compas, 2020) which then lead to more psychological symptoms but also diminished adolescents’ abilities to achieve long-term goals or maintain meaningful relationships, ultimately resulting in low life satisfaction. This may also explain why direct effects of child maltreatment on life satisfaction maintain at discharge, given that inpatient treatment is primarily focused on reducing psychological symptoms and less on improve long-term goal achievement. Thus, future studies might investigate networks of child maltreatment and neurocognitive or emotion regulation processes over longer periods including follow-up assessments after discharge.

Our findings have implications for treatment: Interpersonal symptoms like “never feeling close to others”, “distrust in other people” and “feeling lonely in company of other people” appear repeatedly in pathways from child maltreatment to life satisfaction, especially in pathways involving sexual abuse and physical maltreatment. This finding implicates that these types of maltreatment may interrupt a general sense of trust in other people. Targeting these symptoms in adolescents with high levels of sexual and physical maltreatment experiences may help in experiencing higher life satisfaction in the future. This is underlined by the finding that the association between “distrust in other people” and “worthlessness” disappeared at discharge, suggesting that adolescents reporting sexual abuse may have received corrective experiences in relationships during inpatient treatment. In contrast, emotional abuse and neglect are robustly and more directly connected to life satisfaction at admission and discharge. This may indicate that novel treatments should directly target these child maltreatment experiences, for example using cognitive processing therapy (LoSavio et al., 2021) or narrative exposure therapy (Peltonen & Kangaslampi, 2019) techniques that already showed preliminary efficacy for the treatment of trauma-related symptoms in adolescents.

Several strengths of our study should be noted: We were able to analyze a large sample of adolescents who provided data at both admission and discharge to obtain stable networks at admission and discharge even with many nodes. We combined two network analysis approaches (i.e., regularized partial correlation and Bayesian network analysis) to model both general potential network structures of underlying symptom systems as well as to investigate potentially causal directed pathways from child maltreatment to life satisfaction. This allowed for simultaneous inference on the symptom level compared to previous studies which mainly focused on higher-order constructs. In addition, DAGs estimated with Bayesian network analysis allow to inspect potentially causal pathways between child maltreatment, symptoms and life satisfaction, which is not possible in linear regression models. There are also several limitations to be reported. First, only self-report data were used. We focused on patient self-reports, because we were interested in *subjective* life satisfaction, child maltreatment experiences and psychological symptoms. With the latter being mostly internalizing symptoms, there is evidence that self-reports are sufficiently reliable for assessment (De Los Reyes et al., 2015; Martel et al., 2017; Weitkamp et al., 2013). Despite evidence for discrepancies between self-report and objective recordings (e.g. court proceedings, medical documentations; see Baldwin et al., 2019), recent research indicates a low risk of bias in retrospective self-reports of child maltreatment (Goltermann et al., 2023; Pinto et al., 2014). However, it should be noted that very early experiences of abuse or neglect may not be remembered or may be distorted. Furthermore, a substantial part of the overall sample had to be excluded because of missing data due to technical difficulties during the routine assessment. Nonetheless, comparison between included and excluded adolescents revealed no or negligible differences, indicating that they were mostly comparable. Sample size was substantial compared to other network studies and coefficient stability was obtained. Depressive and eating disorders were the most diagnosed mental disorders in the sample. According to the treatment focus of the center on internalizing mental disorders and especially eating disorders, which both have a higher prevalence in females than in males (Napp et al., 2023; Steffen et al., 2020), female adolescents dominated in the sample. Thus, the results might not be generalizable to males as well as samples with higher proportions of other types than emotional maltreatment, which was highest in our sample. In addition, adolescents who were admitted involuntarily due to suicide attempts or acute crises may show different pathways from child maltreatment to life satisfaction, as they were not included in our sample. Additionally, we used an empirical inclusion criterion for edges in the DAGs (Scutari & Nagarajan, 2013). Therefore, some edges might disappear using more restrictive approaches (e.g., inclusion of edges present in 85% of bootstrapped network samples; Briganti et al., 2021).

In conclusion, we found evidence for direct links from child maltreatment to the HRQoL-related facet of subjective life satisfaction, which were stable from admission to discharge from inpatient treatment in a network analysis of adolescents with mental disorders. Only sexual abuse showed exclusively indirect pathways to life satisfaction through psychological symptoms. This is in line with previous research indicating stronger closeness of sexual abuse to mental disorders compared to other types of child maltreatment. The finding that child maltreatment still directly affects life satisfaction after inpatient treatment further highlights its detrimental effects. This calls for better interventions directly targeting child maltreatment experiences even in the absence of posttraumatic stress disorder, with a focus on emotional neglect due to its linking function between other child maltreatment experiences and life satisfaction. Future studies using network analysis might investigate the interrelations of child maltreatment and basic neurocognitive and emotion regulation processes with life satisfaction in adolescents with and without current mental disorders.

### Electronic Supplementary Material

Below is the link to the electronic supplementary material.

Supplementary Material 1

## Footnotes

1 Of note, we also investigated specific pathways between child maltreatment, life satisfaction and disordered eating symptoms in the subset of patients with eating disorders. These results have been published elsewhere (Monteleone et al., 2023), as they were beyond the scope of this article.

## References

[CR1] Arias, I., Leeb, R. T., Melanson, C., Paulozzi, L. J., & Simon, T. R. (2008). Child maltreatment surveillance; uniform definitions for public health and recommended data elements [Pamphlet (or booklet)]. https://stacks.cdc.gov/view/cdc/11493.

[CR80] Baldwin, J. R., Reuben, A., Newbury, J. B., & Danese, A. (2019). Agreement between prospective and retrospective measures of childhood maltreatment: A systematic review and meta-analysis. *JAMA Psychiatry*, *76*(6), 584–593. 10.1001/jamapsychiatry.2019.0097 10.1001/jamapsychiatry.2019.0097 PMC6551848 30892562

[CR2] Bernstein DP, Stein JA, Newcomb MD, Walker E, Pogge D, Ahluvalia T, Stokes J, Handelsman L, Medrano M, Desmond D, Zule W (2003). Development and validation of a brief screening version of the Childhood Trauma Questionnaire. Child Abuse and Neglect.

[CR3] Borsboom D (2017). A network theory of mental disorders. World Psychiatry.

[CR4] Borsboom D, Cramer AOJ, Schmittmann VD, Epskamp S, Waldorp LJ (2011). The small world of psychopathology. Plos One.

[CR5] Breuer F, Greggersen W, Kahl KG, Schweiger U, Westermair AL (2020). Caught in a web of trauma: Network analysis of childhood adversity and adult mental ill-health. Child Abuse & Neglect.

[CR6] Briganti G, Kornreich C, Linkowski P (2021). A network structure of manic symptoms. Brain Behav.

[CR7] Briganti G, Scutari M, McNally RJ (2022). A tutorial on bayesian networks for psychopathology researchers. Psychological Methods.

[CR8] Bringmann, L., Elmer, T., Epskamp, S., Krause, R., Schoch, D., Wichers, M., Wigman, J., & Snippe, E. (2018). *What do centrality measures measure in psychological networks?*10.13140/RG.2.2.25024.58884. 10.1037/abn0000446 31318245

[CR9] Chen J, Chen Z (2008). Extended bayesian information criteria for model selection with large model spaces. Biometrika.

[CR10] Chen, L. P., Murad, M. H., Paras, M. L., Colbenson, K. M., Sattler, A. L., Goranson, E. N., Elamin, M. B., Seime, R. J., Shinozaki, G., & Prokop, L. J. (2010). Sexual abuse and lifetime diagnosis of psychiatric disorders: systematic review and meta-analysis. Mayo clinic proceedings. 10.4065/mcp.2009.0583 PMC2894717 20458101

[CR11] Cludius B, Mennin D, Ehring T (2020). Emotion regulation as a transdiagnostic process. Emotion.

[CR12] Coghill D, Danckaerts M, Sonuga-Barke E, Sergeant J, Group AEG (2009). Practitioner review: Quality of life in child mental health–conceptual challenges and practical choices. Journal of Child Psychology and Psychiatry.

[CR14] Cohrdes C, Mauz E (2020). Self-Efficacy and Emotional Stability buffer negative effects of adverse childhood experiences on Young Adult Health-Related Quality of Life. Journal of Adolescent Health.

[CR15] Cowell RA, Cicchetti D, Rogosch FA, Toth SL (2015). Childhood maltreatment and its effect on neurocognitive functioning: Timing and chronicity matter. Development and Psychopathology.

[CR16] Cramer AO, Waldorp LJ, van der Maas HL, Borsboom D (2010). Comorbidity: A network perspective. Behavioral and Brain Sciences.

[CR17] Cramer AOJ, van Borkulo CD, Giltay EJ, van der Maas HLJ, Kendler KS, Scheffer M, Borsboom D (2016). Major Depression as a Complex Dynamic System. Plos One.

[CR18] Csardi, G., & Nepusz, T. (2006). The igraph software package for complex network research. *InterJournal*, *Complex Systems*, 1695. https://igraph.org.

[CR19] Davies E, Read J, Shevlin M (2021). The impact of adverse childhood experiences and recent life events on anxiety and quality of life in university students. Higher Education.

[CR20] De Los Reyes A, Augenstein TM, Wang M, Thomas SA, Drabick DAG, Burgers DE, Rabinowitz J (2015). The validity of the multi-informant approach to assessing child and adolescent mental health. Psychological Bulletin.

[CR21] Derogatis LR, Melisaratos N (1983). The brief Symptom Inventory: An introductory report. Psychological Medicine.

[CR22] Dey M, Landolt MA, Mohler-Kuo M (2012). Health-related quality of life among children with mental disorders: A systematic review. Quality of Life Research.

[CR23] Diener E, Emmons RA, Larsen RJ, Griffin S (1985). The satisfaction with Life Scale. Journal of Personality Assessment.

[CR24] Dijkstra EW (1959). A note on two problems in connexion with graphs. Numerische Mathematik.

[CR26] Epskamp S, Cramer AO, Waldorp LJ, Schmittmann VD, Borsboom D (2012). Qgraph: Network visualizations of relationships in psychometric data. Journal of Statistical Software.

[CR25] Epskamp S, Borsboom D, Fried EI (2018). Estimating psychological networks and their accuracy: A tutorial paper. Behavior Research Methods.

[CR27] Fergusson DM, Horwood LJ, Woodward LJ (2000). The stability of child abuse reports: A longitudinal study of the reporting behaviour of young adults. Psychological Medicine.

[CR28] Franke, G. (2000). *Brief Symptom Inventory - Deutsche Version. Manual. Göttingen: Beltz*.

[CR29] Friedman J, Hastie T, Tibshirani R (2008). Sparse inverse covariance estimation with the graphical lasso. Biostatistics.

[CR30] Fritz J, Fried EI, Goodyer IM, Wilkinson PO, van Harmelen AL (2018). A Network Model of Resilience factors for adolescents with and without exposure to Childhood Adversity. Scientific Reports.

[CR31] Glaesmer H, Grande G, Braehler E, Roth M (2011). The German version of the satisfaction with Life Scale (SWLS). European Journal of Psychological Assessment.

[CR32] Goltermann J, Meinert S, Hulsmann C, Dohm K, Grotegerd D, Redlich R, Waltemate L, Lemke H, Thiel K, Mehler DMA, Enneking V, Borgers T, Repple J, Gruber M, Winter N, Hahn T, Brosch K, Meller T, Ringwald KG, Dannlowski U (2023). Temporal stability and state-dependence of retrospective self-reports of childhood maltreatment in healthy and depressed adults. Psychological Assessment.

[CR33] Gruhn MA, Compas BE (2020). Effects of maltreatment on coping and emotion regulation in childhood and adolescence: A meta-analytic review. Child Abuse and Neglect.

[CR34] Hagborg JM, Tidefors I, Fahlke C (2017). Gender differences in the association between emotional maltreatment with mental, emotional, and behavioral problems in Swedish adolescents. Child Abuse & Neglect.

[CR35] Häuser W, Schmutzer G, Brahler E, Glaesmer H (2011). Maltreatment in childhood and adolescence: Results from a survey of a representative sample of the German population. Dtsch Arztebl Int.

[CR36] Herdman M, Rajmil L, Power URSMB (2002). Expert consensus in the development of a European health-related quality of life measure for children and adolescents: A Delphi study. Acta Paediatrica.

[CR37] Jones, P. (2021). *networktools: Tools for Identifying Important Nodes in Networks*. In (Version 1.3.0) https://CRAN.R-project.org/package=networktools.

[CR38] Kaiser, T., Herzog, P., Voderholzer, U., & Brakemeier, E. L. (2021). Unraveling the comorbidity of depression and anxiety in a large inpatient sample: Network analysis to examine bridge symptoms. *Depression and Anxiety*, 38. 10.1002/da.23136. 10.1002/da.23136 33465284

[CR39] Kendler KS, Zachar P, Craver C (2011). What kinds of things are psychiatric disorders?. Psychological Medicine.

[CR40] Klinitzke G, Romppel M, Häuser W, Brähler E, Glaesmer H (2012). [The German version of the Childhood Trauma Questionnaire (CTQ): Psychometric characteristics in a representative sample of the general population]. Psychotherapie, Psychosomatik, Medizinische Psychologie.

[CR41] Lewis SJ, Arseneault L, Caspi A, Fisher HL, Matthews T, Moffitt TE, Odgers CL, Stahl D, Teng JY, Danese A (2019). The epidemiology of trauma and post-traumatic stress disorder in a representative cohort of young people in England and Wales. Lancet Psychiatry.

[CR42] Lorenc T, Lester S, Sutcliffe K, Stansfield C, Thomas J (2020). Interventions to support people exposed to adverse childhood experiences: Systematic review of systematic reviews. BMC Public Health.

[CR43] LoSavio ST, Murphy RA, Resick PA (2021). Treatment outcomes for adolescents versus adults receiving cognitive Processing Therapy for Posttraumatic stress disorder during Community Training. Journal of Traumatic Stress.

[CR44] Macdonald G, Livingstone N, Hanratty J, McCartan C, Cotmore R, Cary M, Glaser D, Byford S, Welton NJ, Bosqui T, Bowes L, Audrey S, Mezey G, Fisher HL, Riches W, Churchill R (2016). The effectiveness, acceptability and cost-effectiveness of psychosocial interventions for maltreated children and adolescents: An evidence synthesis. Health Technology Assessment.

[CR45] Maj M (2018). Why the clinical utility of diagnostic categories in psychiatry is intrinsically limited and how we can use new approaches to complement them. World Psychiatry.

[CR46] Martel MM, Markon K, Smith GT (2017). Research Review: Multi-informant integration in child and adolescent psychopathology diagnosis. Journal of Child Psychology and Psychiatry.

[CR47] McLaughlin KA, Green G, Gruber J, Sampson MJ, Zaslavsky NA, Kessler RC (2012). Childhood adversities and first onset of psychiatric disorders in a national sample of US adolescents. Archives of General Psychiatry.

[CR48] McNally RJ (2016). Can network analysis transform psychopathology?. Behaviour Research and Therapy.

[CR49] McNally RJ, Heeren A, Robinaugh DJ (2017). A bayesian network analysis of posttraumatic stress disorder symptoms in adults reporting childhood sexual abuse. Eur J Psychotraumatol.

[CR51] Monteleone AM, Cascino G, Pellegrino F, Ruzzi V, Patriciello G, Marone L, De Felice G, Monteleone P, Maj M (2019). The association between childhood maltreatment and eating disorder psychopathology: A mixed-model investigation. European Psychiatry.

[CR52] Monteleone AM, Cascino G, Ruzzi V, Pellegrino F, Patriciello G, Barone E, Carfagno M, Monteleone P, Maj M (2021). Emotional traumatic experiences significantly contribute to identify a maltreated ecophenotype sub-group in eating disorders: Experimental evidence. European Eating Disorders Review.

[CR50] Monteleone AM, Cascino G, Meule A, Barone E, Voderholzer U, Kolar DR (2023). Pathways between childhood maltreatment and life satisfaction in adolescents with eating disorders: A network analysis. European Eating Disorders Review.

[CR53] Napp, A. K., Kaman, A., Erhart, M., Westenhöfer, J., & Ravens-Sieberer, U. (2023). Eating disorder symptoms among children and adolescents in Germany before and after the onset of the COVID-19 pandemic. *Frontiers in Psychiatry*, 14. 10.3389/fpsyt.2023.1157402. 10.3389/fpsyt.2023.1157402 PMC10254422 37304440

[CR54] Peltonen K, Kangaslampi S (2019). Treating children and adolescents with multiple traumas: A randomized clinical trial of narrative exposure therapy. Eur J Psychotraumatol.

[CR55] Pinto R, Correia L, Maia A (2014). Assessing the reliability of Retrospective reports of adverse childhood experiences among adolescents with documented childhood maltreatment. Journal of Family Violence.

[CR56] R Core Team. (2022). *R: A language and environment for statistical computing. In (Version 4.2.0)*. R Foundation for Statistical Computing.

[CR57] RStudio Team (2022). *RStudio: Integrated Development for R*. In (Version 2022.07.1) RStudio Inc. https://rstudio.com/.

[CR58] Scutari M (2010). Learning bayesian networks with the bnlearn R Package. Journal of Statistical Software.

[CR59] Scutari M, Nagarajan R (2013). Identifying significant edges in graphical models of molecular networks. Artificial Intelligence In Medicine.

[CR60] Sharpe H, Patalay P, Fink E, Vostanis P, Deighton J, Wolpert M (2016). Exploring the relationship between quality of life and mental health problems in children: Implications for measurement and practice. European Child and Adolescent Psychiatry.

[CR61] Steffen A, Thom J, Jacobi F, Holstiege J, Bätzing J (2020). Trends in prevalence of depression in Germany between 2009 and 2017 based on nationwide ambulatory claims data. Journal of Affective Disorders.

[CR62] Stoltenborgh M, Bakermans-Kranenburg MJ, Alink LRA, van IJzendoorn MH (2015). The prevalence of child maltreatment across the Globe: Review of a series of Meta-analyses. Child Abuse Review.

[CR63] Su, Y., Li, M., D’Arcy, C., Caron, J., & Meng, X. (2023). Childhood maltreatment and major depressive disorder in well-being: A network analysis of a longitudinal community-based cohort. *Psychological Medicine*, 1–9. 10.1017/S0033291723000673. 10.1017/S0033291723000673 PMC10719668 36960542

[CR64] Taillieu TL, Brownridge DA, Sareen J, Afifi TO (2016). Childhood emotional maltreatment and mental disorders: Results from a nationally representative adult sample from the United States. Child Abuse & Neglect.

[CR65] Thapa Bajgain K, Amarbayan M, Wittevrongel K, McCabe E, Naqvi SF, Tang K, Aghajafari F, Zwicker JD, Santana M (2023). Patient-reported outcome measures used to improve youth mental health services: A systematic review. J Patient Rep Outcomes.

[CR66] van Borkulo C, van Bork R, Boschloo L, Kossakowski J, Tio P, Schoevers R, Borsboom D, Waldorp L (2021). Comparing Network structures on three aspects: A permutation test. Psychological Methods.

[CR67] Voderholzer, U., Meule, A., Naab, S., & Kolar, D. R. (in preparation). Misshandlung und Vernachlässigung in der Kindheit bei Jugendlichen in stationärer psychiatrischer Behandlung.

[CR68] Volgenau KM, Hokes KE, Hacker N, Adams LM (2022). A Network Analysis Approach to understanding the relationship between Childhood Trauma and Wellbeing later in Life. Child Psychiatry & Human Development.

[CR69] Weber S, Jud A, Landolt MA (2016). Quality of life in maltreated children and adult survivors of child maltreatment: A systematic review. Quality of Life Research.

[CR70] Weitkamp K, Daniels J, Rosenthal S, Romer G, Wiegand-Grefe S (2013). Health-related quality of life: Cross-informant agreement of father, mother, and self-report for children and adolescents in outpatient psychotherapy treatment. Child Adolesc Ment Health.

[CR71] Wethington HR, Hahn RA, Fuqua-Whitley DS, Sipe TA, Crosby AE, Johnson RL, Liberman AM, Mościcki E, Price LN, Tuma FK, Kalra G, Chattopadhyay SK (2008). The effectiveness of interventions to reduce psychological harm from traumatic events among children and adolescents: A systematic review. American Journal of Preventive Medicine.

[CR72] Widom, C. S. (2014). Longterm Consequences of Child Maltreatment. In J. E. Korbin & R. D. Krugman (Eds.), *Handbook of Child Maltreatment* (pp. 225–247). Springer Netherlands. 10.1007/978-94-007-7208-3_12.

[CR73] Witt A, Brown RC, Plener PL, Brähler E, Fegert JM (2017). Child maltreatment in Germany: Prevalence rates in the general population. Child Adolesc Psychiatry Ment Health.

[CR74] Witt A, Sachser C, Plener PL, Brähler E, Fegert JM (2019). The prevalence and consequences of adverse childhood experiences in the German Population. Dtsch Arztebl Int.

[CR75] World Health Organization (2004). *ICD-10: International Statistical Classification of Diseases and Related Health Problems: Tenth Revision* (2nd ed ed.). World Health Organization. https://apps.who.int/iris/handle/10665/42980.

[CR76] Zbozinek TD, Rose RD, Wolitzky-Taylor KB, Sherbourne C, Sullivan G, Stein MB, Roy-Byrne PP, Craske MG (2012). Diagnostic overlap of generalized anxiety disorder and major depressive disorder in a primary care sample. Depression and Anxiety.

[CR77] Zhao, T., Liu, H., Roeder, K., Lafferty, J., & Wasserman, L. (2011). *High-dimensional Undirected Graph Estimation*. In (Version R package version 1.3.5) https://CRAN.R-project.org/package=huge. PMC4729207 26834510

[CR78] Zhou J, Fan A, Zhou X, Pao C, Xiao L, Feng Y, Dong B (2022). Interrelationships between childhood maltreatment, depressive symptoms, functional impairment, and quality of life in patients with major depressive disorder: A network analysis approach. Child Abuse and Neglect.

[CR79] Zipfel S, Herzog W, Kruse J, Henningsen P (2016). Psychosomatic medicine in Germany: More timely than ever. Psychotherapy and Psychosomatics.

