# Supplementary material for: Pathways between Child Maltreatment, Psychological Symptoms, and Life Satisfaction: A Network Analysis in Adolescent Inpatients
Source: Res Child Adolesc Psychopathol. 2024 Jan 30;52(6):969–82. doi: 10.1007/s10802-024-01172-2 (PMC11108895; doi:10.1007/s10802-024-01172-2)
Supplement: Supplementary file 1 — Supplementary Material 1 [file 10802_2024_1172_MOESM1_ESM.docx]

**Pathways between child maltreatment, psychological symptoms, and life satisfaction: a network analysis in adolescent inpatients – supplementary materials**

David R. Kolar, Alessio Maria Monteleone, Giammarco Cascino, Sebastian Ertl, Adrian Meule, Silke Naab, Ulrich Voderholzer

**all correspondence to** [**david.kolar@ur.de**](mailto:david.kolar@ur.de)

**Supplementary materials – all correspondence to** [**david.kolar@ur.de**](mailto:david.kolar@ur.de)

# Table S1. Differences between diagnostic groups on key measures.

Table S1 provides mean and standard deviation of key measures at admission of diagnostic groups.

|  | Affective disorders | | | Anxiety disorders | | | Obsessive–  compulsive disorder | | | Anorexia nervosa | | | Bulimia nervosa | | |  |  |  |
| --- | --- | --- | --- | --- | --- | --- | --- | --- | --- | --- | --- | --- | --- | --- | --- | --- | --- | --- |
| At admission | *N* | *M* | *SD* | *N* | *M* | *SD* | *N* | *M* | *SD* | *N* | *M* | *SD* | *N* | *M* | *SD* | *F* | *df* | *p* |
| Age | 322 | 16.22^a^ | 1.12 | 57 | 16.07 | 0.94 | 70 | 16.33 | 1.42 | 375 | 15.78^a.b^ | 1.33 | 72 | 16.11^b^ | 1.12 | 6.48 | 4, 194.38 | <.001 |
| Length of stay | 322 | 77.94^ab^ | 45.40 | 57 | 85.26^c^ | 47.04 | 70 | 98.11^a^ | 42.84 | 375 | 102.96^bc^ | 53.09 | 72 | 94.78 | 50.35 | 12.06 | 4, 192.52 | <.001 |
| CTQ emotional abuse | 322 | 10.74^abc^ | 5.14 | 57 | 8.32^ad^ | 4.04 | 70 | 8.11^be^ | 4.05 | 375 | 8.52^c^ | 3.91 | 72 | 10.50^de^ | 5.13 | 13.03 | 4, 189.90 | <.001 |
| CTQ emotional neglect | 322 | 10.96^ab^ | 4.38 | 57 | 10.37^c^ | 4.29 | 70 | 8.31^acd^ | 3.47 | 375 | 9.37^b^ | 4.02 | 72 | 10.49^d^ | 4.88 | 10.29 | 4, 190.68 | <.001 |
| CTQ physical abuse | 322 | 5.89^ab^ | 2.10 | 57 | 5.53 | 1.80 | 70 | 5.40^a^ | 1.68 | 375 | 5.34^bc^ | 1.24 | 72 | 5.85^c^ | 1.73 | 5.15 | 4, 182.94 | <.001 |
| CTQ physical neglect | 322 | 6.82^ab^ | 2.26 | 57 | 6.88^c^ | 2.23 | 70 | 5.96^ac^ | 1.59 | 375 | 6.36^b^ | 2.03 | 72 | 6.83 | 2.63 | 4.72 | 4, 191.83 | .001 |
| CTQ sexual abuse | 322 | 6.00^ab^ | 2.76 | 57 | 5.39 | 2.20 | 70 | 5.23^a^ | 0.87 | 375 | 5.21^b^ | 1.17 | 72 | 5.68 | 2.24 | 6.34 | 4, 190.68 | <.001 |
| CTQ total score | 322 | 40.41^abc^ | 12.38 | 56 | 36.64^a^ | 10.95 | 69 | 32.94^bd^ | 8.57 | 371 | 34.86^ce^ | 9.43 | 69 | 39.41^de^ | 12.82 | 14.53 | 4, 185.19 | <.001 |
| BSI GSI | 322 | 1.65^ab^ | 0.68 | 57 | 1.43 | 0.67 | 70 | 1.21^ac^ | 0.72 | 375 | 1.29^bd^ | 0.71 | 72 | 1.59^cd^ | 0.75 | 14.31 | 4, 189.29 | <.001 |
| SWLS total score | 322 | 7.59^a^ | 6.65 | 57 | 8.06^b^ | 6.99 | 70 | 8.09^c^ | 7.57 | 375 | 6.76^d^ | 6.94 | 72 | 4.79^abcd^ | 5.69 | 4.09 | 4, 191.19 | .003 |

Note: Differences between diagnostic groups are also displayed as obtained by Welch’s ANOVA and Games-Howell post-hoc comparisons. Statistically significant group differences between diagnostic groups are displayed by shared superscript letters. BSI GSI: Brief Symptom Inventory, Global Severity Index; CTQ: Childhood Trauma Questionnaire; SWLS: Satisfaction With Life Scale.

# Table S2. Comparison of admission (composite) scale scores between patients showing without missing and those with missing BSI values at discharge

|  | No missing values at discharge | | | Missing values at discharge | | |  |  |  |  |
| --- | --- | --- | --- | --- | --- | --- | --- | --- | --- | --- |
| Measure (at admission) | *N* | *M* | *SD* | *N^a^* | *M* | *SD* | *t* | *df* | *p* | *d* |
| BSI 30 item mean score | 643 | 1.73 | 0.82 | 253^b^ | 1.86 | 0.80 | -2.05 | 472.18 | .04 | -0.15 |
| SWLS total score | 652 | 15.36 | 6.16 | 244^c^ | 14.86 | 6.59 | 1.02 | 411.25 | .31 | – |
| CTQ total score | 635 | 7.28 | 2.13 | 261^d^ | 7.78 | 2.48 | -2.84 | 425.81 | .005 | -0.22 |

Note: ^a^Participants are not mutually exclusive. ^b^Participants missing at least one of the 30 BSI items at discharge. ^c^Participants missing at least one SWLS item at discharge. ^d^Participants that were excluded at discharge due to at least one missing item of the BSI or SWLS items that were included in the network analyses. Cohens *d* was calculated with the mean standard deviation as used in Welch’s *t*-test; BSI 30 item mean score: Brief Symptom Inventory mean item score based on the 30 items that were selected for inclusion in the network analysis; CTQ: Childhood Trauma Questionnaire; SWLS: Satisfaction With Life Scale.

# Table S3. Excluded Items from the BSI-53 according to three criteria.

| **Item** | **Item name** | **Item description** | **Exclusion criteria** |
| --- | --- | --- | --- |
| bsi02 | faint | Faintness or dizziness | b |
| bsi03 | thoughtcontr | Idea someone controls your thoughts | c |
| bsi04 | blame | Others blame you for your troubles | c |
| bsi05 | memory | Difficulties remembering | a |
| bsi07 | chestpain | Chest pain | b |
| bsi08 | agoraphobia | Afraid in open spaces | a,c |
| bsi12 | startle | Easily startled | c |
| bsi20 | feelhurt | Feelings easily hurt | a |
| bsi26 | checking | Checking behaviors | c |
| bsi28 | claustrophobia | Afraid to travel in bus, subway, train | c |
| bsi29 | breathing | Trouble breathing | a, c |
| bsi31 | avoidfear | Avoiding things due to fear | c |
| bsi32 | blackout | Mind going blank | a |
| bsi33 | numb | Numbness of body parts | b |
| bsi34 | punished | Should receive punishment for sins | c |
| bsi40 | urgeharm | Urges to harm someone | c |
| bsi41 | urgebreak | Urges to break things | c |
| bsi42 | selfconscious | Feeling self-conscious with others | a |
| bsi45 | panic | Panic or terror | c |
| bsi46 | arguments | Frequent arguments | c |
| bsi47 | nervousalone | Nervous when being alone | a |
| bsi48 | credit | Not receiving credit for achievements | c |
| bsi51 | takenadvantage | Being taken advantage of | a |

Note: Symptoms were measured at admission and discharge. To reduce the number of items for the network analysis, items were excluded based on three criteria: a) theoretical importance in adolescence, b) collinearity of items at admission as identified with the *goldbricker* function of the *networktools* package, version 1.3.0 (Jones, 2021) and c) by inspecting histograms and removing items that were of less importance and not frequently endorsed (Fig. 1).

# Figure S1. Histograms of all BSI-53 items.


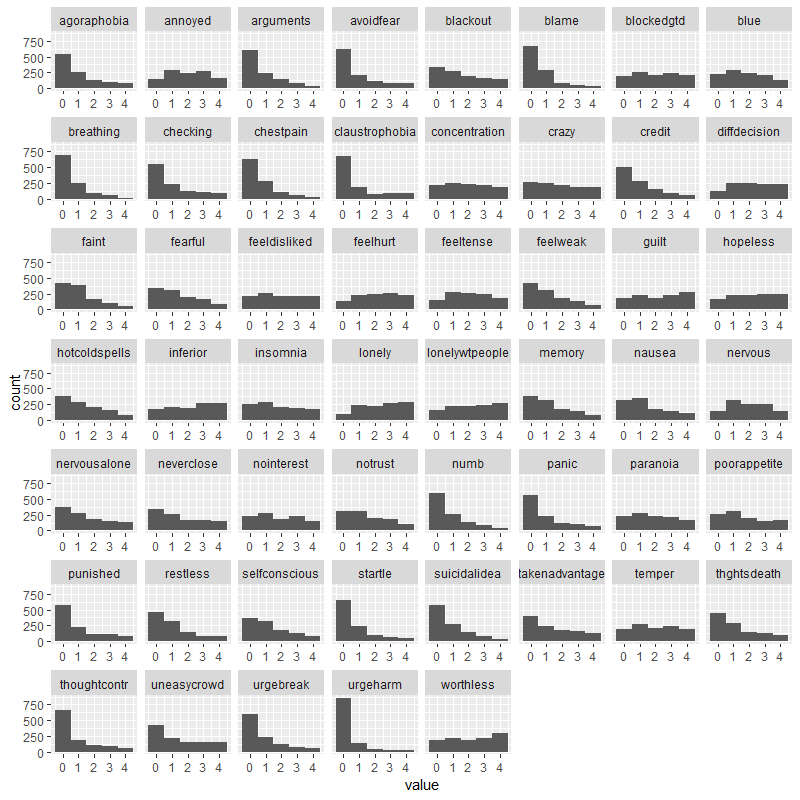


# Table S4. Pre-post treatment comparisons on psychopathology and life satisfaction for all participants.

|  | Admission | | | Discharge | | |  |  |  |  |
| --- | --- | --- | --- | --- | --- | --- | --- | --- | --- | --- |
| Measure | *N* | *M* | *SD* | *N* | *M* | *SD* | *t* | *df* | *p* | *d* |
| BSI-53 GSI | 1104 | 1.44 | 0.72 | 937 | 1.02 | 0.71 | 16.02 | 654 | <.001 | 0.63 |
| SWLS | 1109 | 6.32 | 6.39 | 938 | 7.06 | 6.99 | -9.02 | 654 | <.001 | 0.35 |

Note: BSI-53 GSI: global severity index of the Brief Symptom Inventory-53; SWLS: Satisfaction With Life Scale.

# Figure S2. Directed acyclic graphs (DAGs) showing all pathways from CTQ nodes to SWLS nodes for admission (A) and discharge (B).

| A | B |
| --- | --- |
| 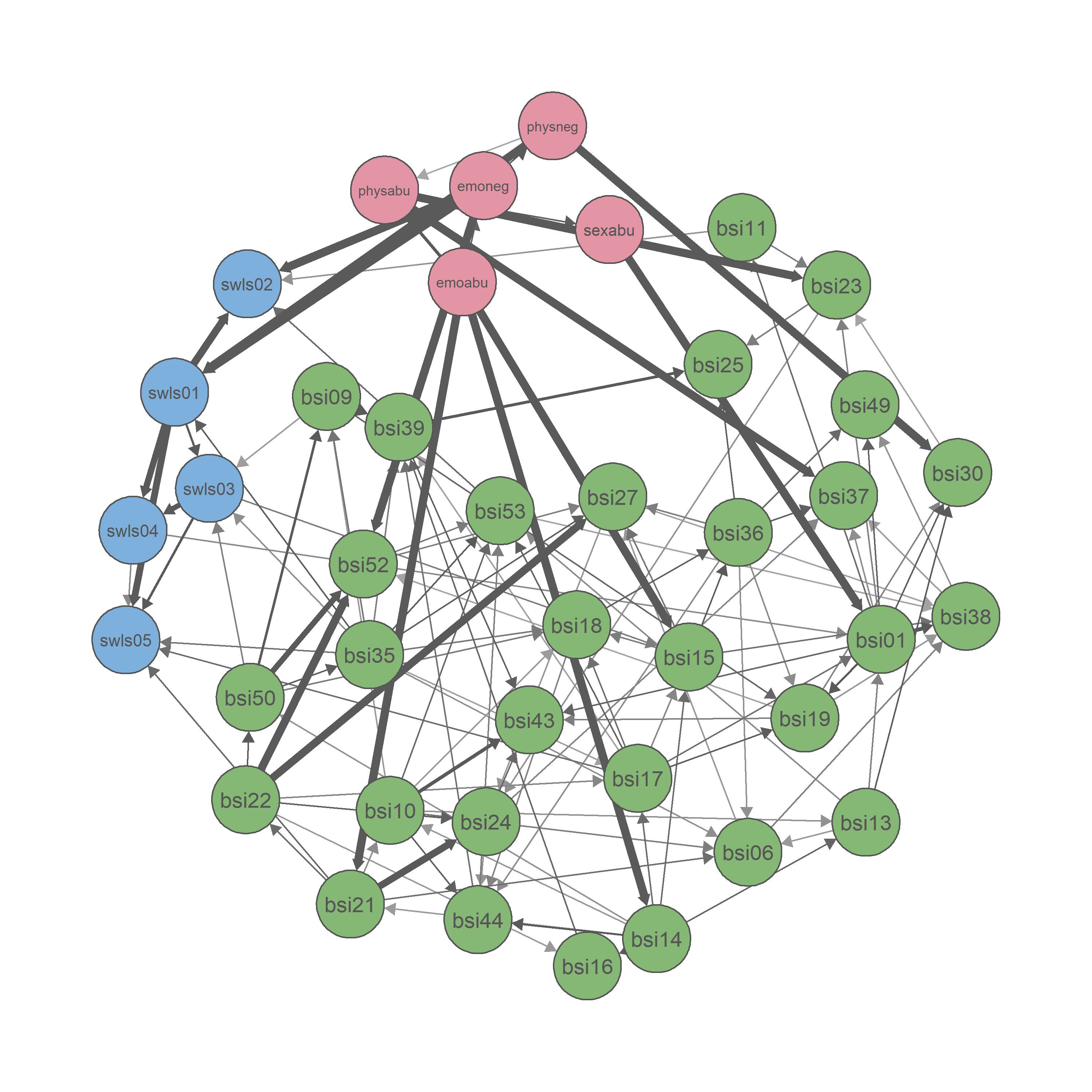 | 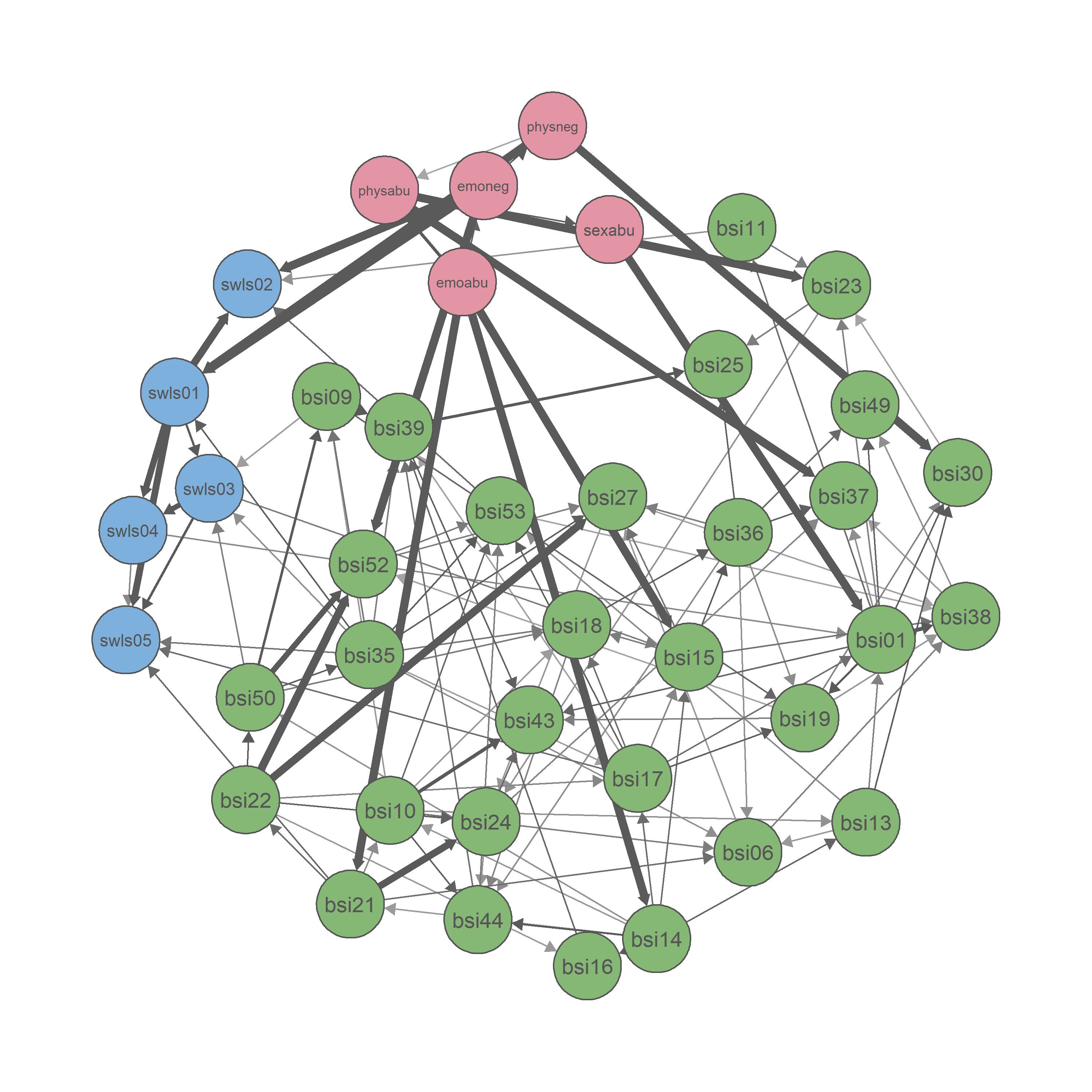 |
